# The Vaccination Question in the Light of Modern Experience. An Appeal for Reconsideration

**Published:** 1915-06

**Authors:** 


**The Vaccination Question in the Light of Modern Experience. An Appeal for Reconsideration.** By C. Killick Millard, M.D., D.Sc. Pp. xviii., 243. London: H. K. Lewis. 1914. Price, 6s. net.

It 's a long way back to one's school days, but one can just remember

> "Mons parturibat, gemitus immanes ciens
> Eratque in terris maxima expectatio
> At ille murem peperit."

Dr. Millard is patently ingenuous, and has succeeded in proving at some length what no one of experience denies: that vaccination, while it protects the individual for a time, needs repetition to maintain its power of protection, otherwise modified cases occur in the once-vaccinated which may be unrecognised, and thus act as centres of infection. Hence he argues that it were better to deal with *unvaccinated* populations, as then the majority of cases would be clinically recognisable, and the disease more readily controlled. We trust we have not misinterpreted his thesis, and may leave him, until the Day, to enjoy his belief, which we do not share.

